# Maximizing biodiesel yield of a non-edible chinaberry seed oil via microwave assisted transesterification process using response surface methodology and artificial neural network techniques

**DOI:** 10.1016/j.heliyon.2023.e22031

**Published:** 2023-11-08

**Authors:** Rehman Akhtar, Ameer Hamza, Luqman Razzaq, Fayaz Hussain, Saad Nawaz, Umer Nawaz, Zara Mukaddas, Tahir Abbas Jauhar, A.S. Silitonga, C Ahamed Saleel

**Affiliations:** a Department of Mechanical Engineering Technology, University of Gujrat, 50700, Pakistan; b Modeling Evolutionary Algorithms Simulation and Artificial Intelligence, Faculty of Electrical & Electronics Engineering, Ton Duc Thang University, Ho Chi Minh City, Viet Nam; c Department of Mechanical, Mechatronic and Manufacturing Engineering, University of Engineering & Technology, Lahore (New Campus), KSK, Sheikhupura, 39350, Pakistan; d Department of Chemistry, University of Gujrat, 50700, Pakistan; e Centre for Technology in Water and Wastewater, School of Civil and Environmental Engineering, Faculty of Engineering and Information Technology, University of Technology Sydney, NSW, 2007, Australia; f Center of Renewable Energy, Department of Mechanical Engineering, Politeknik Negeri Medan, 20155, Medan, Indonesia; g Department of Mechanical Engineering, College of Engineering, King Khalid University, Asir, Abha, 61421, Saudi Arabia

**Keywords:** Biodiesel, Chinaberry seed oil, Response surface methodology, Artificial neural network

## Abstract

In this study, the non-edible Chinaberry Seed Oil (CBO) is converted into biodiesel using microwave assisted transesterification. The objective of this effort is to maximize the biodiesel yield by optimizing the operating parameters, such as catalyst concentration, methanol-oil ratio, reaction speed, and reaction time. The designed setup provides a controlled and effective approach for turning CBO into biodiesel, resulting in encouraging yields and reduced reaction times. The experimental findings reveal the optimal parameters for the highest biodiesel yield (95 %) are a catalyst concentration of 1.5 w/w, a methanol-oil ratio of 6:1 v/v, a reaction speed of 400 RPM, and a reaction period of 3 min. The interaction of the several operating parameters on biodiesel yield has been investigated using two methodologies: Response Surface Methodology (RSM) and Artificial Neural Network (ANN). RSM provides better modeling of parameter interaction, while ANN exhibits lower comparative error when predicting biodiesel yield based on the reaction parameters. The percentage improvement in prediction of biodiesel yield by ANN is found to be 12 % as compared to RSM. This study emphasizes the merits of both the approaches for biodiesel yield optimization. Furthermore, the scaling up this microwave-assisted transesterification system for industrial biodiesel production has been proposes with focus on its economic viability and environmental effects.

## Highlights

• The maximum biodiesel yield of 95 % was obtained.
• The biodiesel yield predicted by ANN model was close to the experimental optimized yield.
• The R^2^ value was close to one for the training and was 0.944 for validation.
• RSM better described the interactions of individual parameters and ANN worked better for overall yield prediction.

## Nomenclature

CBO: Chinaberry seed oil
CBOME: Chinaberry oil methyl ester
RSM: Response surface methodology
ANN: Artificial neural networks
AV: Acid Value
FFA: Free fatty acid
CV: Calorific Value
HSD: High speed diesel
GCMS: Gas chromatography mass spectrum
R^2^: Coefficient of determination
RASE: Root average squared error
MAD: Mean absolute deviation
SSE: Sum of squares error

## 1 Introduction

The world is currently experiencing a historic energy crisis because of the diminishing supply of fossil fuels, over-reliance on them, and their rising depletion [1]. As a result, there have been more greenhouse gas emissions, hotter weather, and numerous other environmental problems [2]. Environmental health concerns are greatly impacted by the growing carbon footprint, notably that of the automotive industry and other associated industries [3]. To diversify the renewable fuels used in automobiles, efforts are being made. Numerous scientific studies have shown that these global problems are primarily the result of human activity [4]. Indeed, the rapid expansion of polluting industries, the rapid expansion of the transportation sector, and excessive energy consumption have all made a significant contribution to the natural resources’ depletion and environmental degradation through the release of greenhouse gases, particularly CO_2_, which is the primary cause of climate change. This leads to global warming [5]. Therefore, it is crucial to create substitution by sustainable energy sources. One such choice is biodiesel, a sustainable, clean-burning fuel that can be used in diesel engines and is generated from vegetable or animal-based sources. Biodiesel has drawn a lot of interest as a potential substitute for fossil fuels as it is advantageous to the environment and economy [6]. Compared to normal petroleum-based diesel fuel, it generates fewer greenhouse gases, is non-toxic, and biodegradable [7].

The adaptability of biodiesel's feedstocks is one of its main advantages [8]. Both edible and non-edible oils can be used to make biodiesel, making it a desirable option for nations with an abundance of non-edible oilseeds [9]. One such underutilized resource that can be utilized to make biodiesel is the non-edible oilseed known as chinaberry seed. The seeds of the Asiatic and African Chinaberry tree are used to make China-Berry seed oil (CBO). Non-edible oils are frequently more challenging to turn into biodiesel because of their higher acid value, higher moisture content, and higher level of impurities [10]. Therefore, it's crucial to maximize the amount of biodiesel that can be produced from CBO.

Vegetable oils can be transformed into biodiesel using the inventive and effective process of microwave-assisted transesterification [11]. The most popular method for manufacturing biodiesel in laboratory and commercial settings with affordable and environmentally responsible catalysts is transesterification [12]. In this method, the reaction mixture is heated using microwaves, accelerating the reaction rate, cutting the reaction time, and increasing the production of biodiesel. For transesterification, microwaves have a number of advantages over conventional methods, including higher yields, a need for less energy, and the creation of less wastes [13].

For statistical analysis to determine the importance of the supplied data, RSM was utilized [14]. A statistical technique for enhancing the process parameters of chemical reactions is called Response Surface Methodology (RSM) [15]. To create the best possible operating circumstances, RSM creates an appropriate experimental design model [16]. RSM provides a number of benefits, including the ability to simultaneously analyze many components and their interactions, minimize the number of tests required, and model intricate correlations between variables [17]. RSM is a crucial tool for R&D as it facilitates to optimize for goods and processes effectively and efficiently, which reduces costs and produces better results [18]. The yield optimization of biodiesel from a range of feedstock, including non-edible oils, has been accomplished with the help of RSM.

RSM has proven effective in modeling parameters, but in recent times, machine learning approaches, particularly Artificial Neural Networks (ANNs), have gained popularity for predicting product outcomes based on given parameters [19]. ANNs excel in modeling complex functions with higher accuracy compared to RSM, as RSM is limited to quadratic behavior of the parameters [20]. This advantage positions ANNs as a more competent candidate for modeling and optimizing the given phenomenon.

The process of transesterification with microwave assistance was studied by the researchers [21,22]. They used microwave ovens as the reactor and used cooking oil as the raw material for the transesterification process. According to the study, microwave-assisted transesterification can produce up to 96 % more biodiesel than traditional methods can. The process was also shown to be quicker, more energy-efficient, and less wasteful. The team's results have significant ramifications for the creation of more economical and environmentally friendly biodiesel manufacturing systems. A recent study by Luqman et al. [23] was released, employing palm and cotton seed oil as the fuel and microwave-assisted transesterification to produce biodiesel. They found that their approach reduced reaction time and energy usage while producing more biodiesel than conventional approaches.

This present study is an effort to convert CBO into biodiesel with optimized yield. Microwave assisted transesterification process has been used for the conversion of CBO into biodiesel. Microwave assisted transesterification process is a tangible solution over the conventional transesterification process in terms of energy consumption. In the setup for microwave-assisted transesterification, a reactor vessel, a condenser, and a stirrer are frequently employed. The reactor vessel's mixture of CBO, methanol, and a catalyst is heated using microwaves. Regular measurements of the acid value and biodiesel output are used to track the reaction's progress. The resulting biodiesel is then cleaned, dried, and refined to get rid of any impurities. The variations in biodiesel yield are observed owing to the operating parameters of the transesterification process. An interaction among these parameters is developed by RSM and biodiesel yield predictions are made. Another technique ANN has been used in this study to predict biodiesel yield. It has been found that the predicted results of ANN are more precise and very near to experimental results.

## 2 Materials and methods

### 2.1 Materials

A microwave oven, a reaction vessel, a mechanical overhead stirrer, and a condenser comprised the microwave-assisted transesterification system. A 250 mL round bottom flask with a reflux condenser was used as the reaction vessel. CBO was purchased from a local market of Pakistan. The other chemicals such as the alcohol and catalyst, respectively, were purchased from the Sigma Aldrich. The impurity of methanol and KOH was 99.9 % and 85 % respectively.

### 2.2 Biodiesel production

The free fatty acid value (FFA) is the determining step in biodiesel production from any feedstock. Before converting CBO into biodiesel using mineral acids, the acid value (AV) should be reduced. CBO's AV was 3.78, which was higher than the average AV. Therefore, the free fatty acid (FFA) content of the raw CBO was reduced by esterifying it with mineral acids (H_2_SO_4_ and CH_3_OH), which in turn determined the AV.

The amount of methanol used was the most crucial element in the esterification process. FFA reduction would be more efficient if there was more methanol in the mixture. Other factors included the reaction's 600 RPM speed, 60 °C temperature, and 3 h of reaction time. Equation (1) was used to calculate the catalyst dosage for transesterification.

(1) $$Catalyst\;amont=\frac{Catalyst\;concentration\times Amount\;of\;CBO\;used}{100}$$

**Fig. 1:**
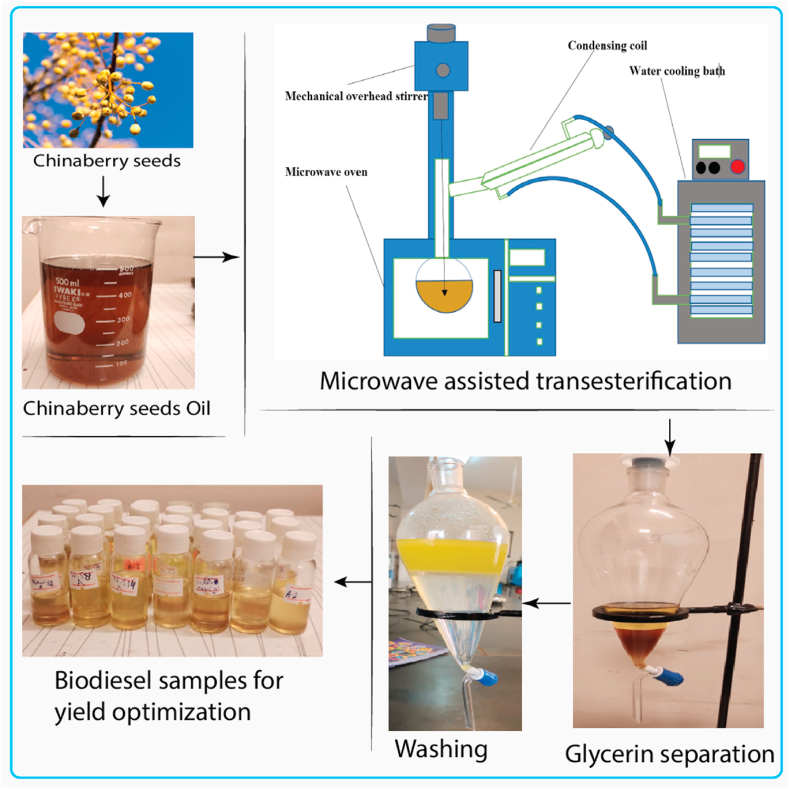
Process flow diagram.

Microwave assisted transesterification has been used to transform CBO into biodiesel. The KOH catalyst in the presence of methanol were used. In the presence of KOH, methanol was added to CBO at reaction speeds ranging from 100 to 400 RPM for durations of 1–3 min before settling overnight. Glycerin, being heavier, settled down in the bottom layer and looked to be collected in the top layer; the latter was separated using a separating funnel. To remove contaminants like catalysts and unused methanol, *trans*-esterified biodiesel was continually washed in hot water. Biodiesel's washing required distilled water, and the process was repeated until the utilized distilled water was transparent. The whole process flow diagram has been shown in Fig. 1. A rotary evaporator was used to remove the remaining methanol and water from the biodiesel. Biodiesel yield was calculated using Equation (2) [24]:

(2) $$Yield=\frac{Amount\;of\;biodiesel\;produced}{Amount\;of\;CBO\;used}\times 100$$

### 2.3 Biodiesel characteristics analysis

Using a bomb calorimeter, the calorific value of biodiesel was calculated. The Cleveland open cup apparatus (Koehler, New York, NY, USA) was used to measure the flashpoint of biodiesel. The GCMS-QP2010 plus was used to determine the FAME composition. The carrier gas was helium gas. The determination of acid value required titration of CBO with a combination of 0.5 N KOH and 50 mL of distilled water. As an indication, 0.25 g of phenolphthalein and 25 mL of ethyl alcohol were combined. A 50 mL solution (95 % ethyl alcohol and 5 % distilled water) was made, and 1 mL of an indicator was then added to a CBO solution. The AV of WCO was calculated using Equation (3) [24]:

(3) $$Acid\;Value=\frac{56.1\times N\times V}{W}$$

Titration (4) [24],

(4) $$FFA=\frac{AV}{2}$$

where,

N: Normality of KOHV: Volume of KOH and distilled water used for titration W: Weight of CBO used.

### 2.4 Method for the biodiesel yield optimization

The catalyst concentration, the methanol to oil ratio, the reaction speed, and the time were the four main operating factors that influence biodiesel yield. For the purpose of optimizing biodiesel yield, experimental conditions were created using JMP Pro 16 software. The operating parameters with their corresponding ranges have been shown in the Table 1.

**Table 1:** Process parameters for yield optimization.

Operating Parameters	Units	Range
Reaction Speed	RPM	100–400
Reaction Time	Mint	1–3
Catalyst Concentration	w/w	0.2–1.5
Methanol to oil ratio	v/v	6–12

**Fig. 2:**
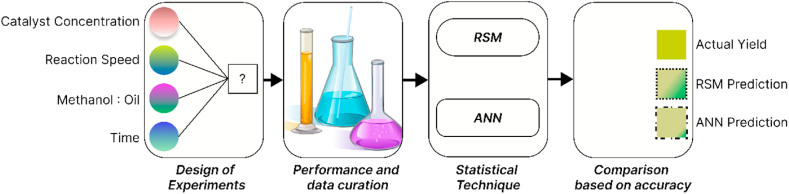
Flow of different stages.

On JMP Pro 16, the data gathered from experiments were analyzed and then interpreted. Regression analysis, response surface mapping, and analysis of variance (ANOVA) are the three primary analytical stages needed to create optimal circumstances. Artificial Neural Network (ANN) was also applied to predict the biodiesel yield. The experimentally optimized biodiesel yield was compared for RSM and ANN to find the merits of the two techniques for yield prediction and optimization. The flow of different stages is shown in Fig. 2.

## 3 Result and analysis

### 3.1 Characterization of biodiesel

Table 2 describes the physical and chemical characteristics of CBO sourced biodiesel. These characteristics have been contrasted with typical biodiesel thermophysical characteristics according to ASTM standards. The components of FAME have been identified by GCMS; Table 3 shows the percentage composition of the various long carbon chain constituents.

**Table 2:** Physical and chemical characteristics of biodiesel.

Properties	Diesel	CBOME
Density @ 15 °C (g/cm^3^)	0.8310	0.9278
Viscosity @ 40 °C (mm^2^/s)	3.9015	4.5250
Acid Value (mg KOH/g oil)	<0.245	0.87
Flash Point (°C)	79	133.5
Calorific Value (MJ/kg)	42	32.80
Pour Point (°C)	7	−28

**Table 3:** GCMS analysis of biodiesel sourced from the CBO.

Chemical Name	Chemical Formula	CBOME
Palmitic Acid	C16:0	9.5
Palmitoleic Acid	C16:1	1.2
Stearic Acid	C18:0	4.1
Oleic Acid	C18:1	59.5
Linoleic Acid	C18:2	11.31
Linolenic Acid	C18:3	1.05
Methyl Arachidate	C20:0	8.28
Methyl Erucate	C22:1	5.06

### 3.2 Biodiesel yield optimization

**Fig. 3:**
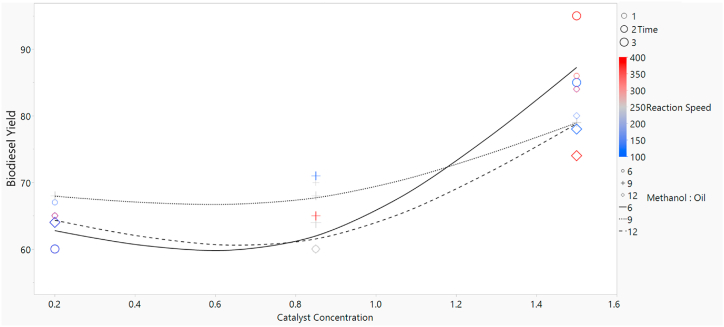
Biodiesel yield against different reaction parameters.

With the design of experiments, a total of 26 experiments were performed. Fig. 3 shows the biodiesel yield against different reaction parameters. The size of shape corresponds to the time, the colors red, gray, and blue represent the reaction speeds and the markers circle, plus and diamond signify the methanol to oil ratio. The lines solid, short dash and long dash are the trend fits to the data. The graph between catalyst concentration and biodiesel yield is formed.

From the Table 4, the biodiesel yield increases with an increase in catalyst concentration. However, at low catalyst concentration values, the higher yield is obtained at 250 RPM, 1 min reaction time and 9 v/v methanol-oil ratio. As the catalyst concentration is increased, the maximum yield behavior is changed. Now the values for time, reaction speed and methanol to oil ratio came to be 2 mint, 100 RPM and 9 v/v respectively. The yield for the methanol to oil ratio 6 v/v first decreases then increased to maximum value of 95 % for catalyst concentration = 1.5w/w, reaction time = 3 min and reaction speed = 400 RPM.

**Table 4:** Interaction among the operating parameters and actual and predicted yield from experimental and RSM.

Run	Catalyst Concentration	Reaction Speed	Methanol: Oil	Reaction Time	Actual Yield	Predicted Yield
	w/w	RPM	v/v	minute	(%)	(%)
1	1.5	100	12	1	80	79.5
2	0.85	250	9	3	64	66.5
3	0.2	400	6	3	60	62.5
4	0.85	250	12	2	60	59.1
5	0.85	250	9	2	68	65.9
6	0.85	400	9	2	65	68.8
7	1.5	400	12	1	84	80.9
8	0.85	250	9	1	70	68.9
9	0.2	400	6	1	65	64.3
10	0.85	250	6	2	60	62.5
11	0.2	400	12	3	64	61.2
12	0.2	400	12	1	65	66.4
13	0.85	250	9	2	68	65.9
14	0.2	100	6	1	67	64.5
15	0.2	100	12	3	64	63.5
16	1.5	250	9	2	79	83.5
17	1.5	400	12	3	74	78.7
18	1.5	400	6	1	86	88.4
19	1.5	100	6	1	84	84.8
20	1.5	100	12	3	78	76.8
21	0.2	250	9	2	68	64.9
22	0.85	100	9	2	71	68.5
23	1.5	400	6	3	95	88.8
24	1.5	100	6	3	85	85.2
25	0.2	100	6	3	60	61.8
26	0.2	100	12	1	65	69.2

### 3.3 Validation of optimized techniques

**Fig. 4:**
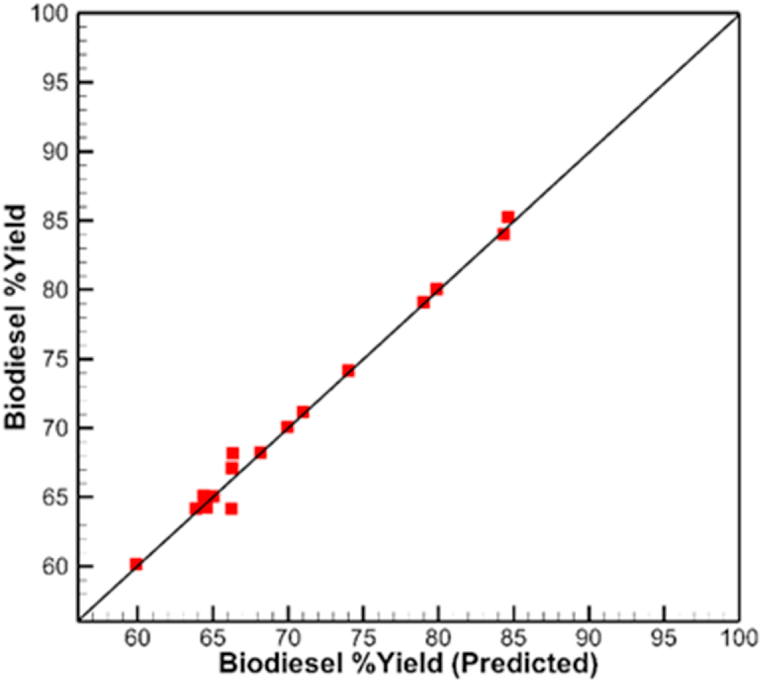
RSM actual vs. predicted yield for biodiesel.

As the biodiesel yield mostly depends on these operating parameters, RSM develops an interaction between them in the transesterification process [25]. Therefore, at optimal operating parameters, the biodiesel yield would be optimal. Consider the following four input reaction variables: catalyst concentration (C), reaction time (A), methanol to oil ratio (B), and reaction speed (D). For 26 experiments, the yield of CBO biodiesel was achieved. Fig. 4 shows the relation between RSM prediction and actual yields.

The impressive consistency between the experimental and expected biodiesel output is shown by the linear regression of fit. The obtained biodiesel yields varied from 60 % to 95 %. Additionally, the magnitude importance of each term in the model was established. Every factor, including those with linear, quadratic, and interaction effects, has a significant impact on the production of biodiesel. As can be seen, catalyst concentration had the biggest linear impact on biodiesel yield among the four factors considered. It has a more significant effect than the other factors, which include reaction time, the methanol-oil ratio, and stirring speed. However, the quadratic components for all three variables—catalyst concentration, methanol-oil ratio, and stirring speed—have a greater effect than their linear equivalents. Most of the literature claims that the methanol-oil ratio and catalyst concentration have the highest effects on biodiesel yield. Table 5 shows the analysis of variance.

**Table 5:** Analysis of variance.

Source	Nparm	DF	Sum of Squares	F Ratio	Prob > F
Catalyst Concentration (0.2,1.5)	1	1	1549.3889	89.9996	<.0001*
Reaction Speed (100,400)	1	1	0.8889	0.0516	0.8244
Methanol: Oil (6,12)	1	1	43.5556	2.5300	0.1400
Time (1,3)	1	1	26.8889	1.5619	0.2373
Catalyst Concentration*Reaction Speed	1	1	12.2500	0.7116	0.4169
Catalyst Concentration*Methanol: Oil	1	1	100.0000	5.8087	0.0346*
Reaction Speed*Methanol: Oil	1	1	6.2500	0.3630	0.5590
Catalyst Concentration*Time	1	1	9.0000	0.5228	0.4847
Reaction Speed*Time	1	1	0.2500	0.0145	0.9063
Methanol: Oil*Time	1	1	9.0000	0.5228	0.4847
Catalyst Concentration*Catalyst Concentration	1	1	174.0047	10.1074	0.0088*
Reaction Speed*Reaction Speed	1	1	19.2669	1.1192	0.3128
Methanol: Oil*Methanol: Oil	1	1	70.7791	4.1114	0.0675
Time*Time	1	1	7.7791	0.4519	0.5153

### 3.4 Effect of operating parameters

**Fig. 5:**
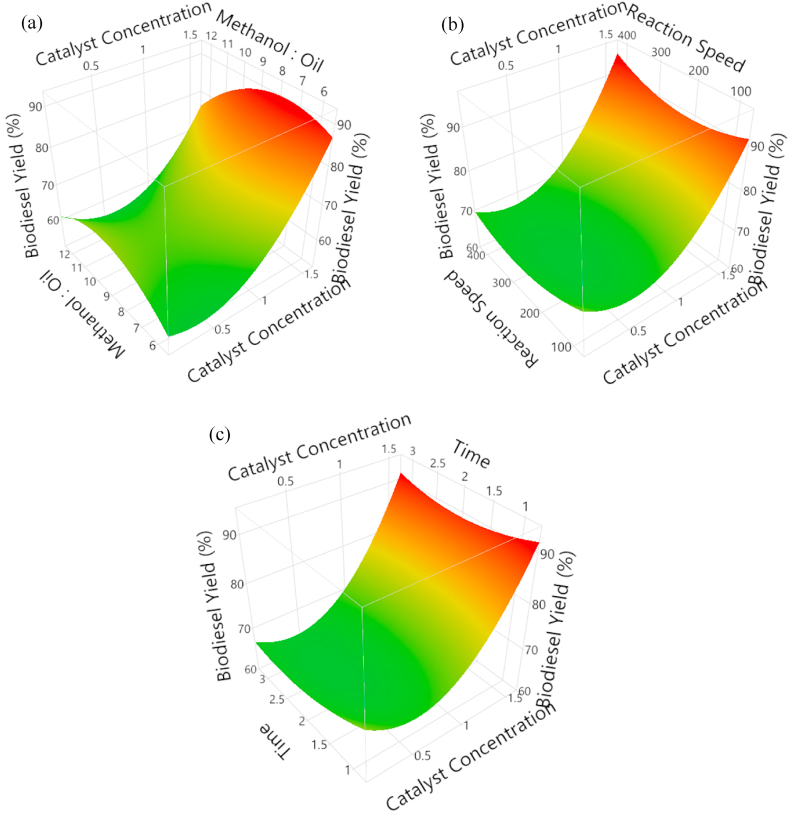
Experimentally obtained RSM plot to investigate the effect of **(a)** methanol to oil ratio **(b)** stirring speed **(c)** reaction time at a constant catalyst concentration.

This section describes how the biodiesel yield is affected by different process parameters. To examine the impact of catalyst concentration, methanol-oil ratio, stirring speed, and reaction time, Fig. 5a shows an experimentally obtained RSM plot. A range of catalyst concentrations, from 0.2 w/w to 1.5 w/w, were used. As seen in Fig. 2a, the production of biodiesel increased after it climbed from 0.9w/w to 0.2w/w. By raising the methanol to oil ratio from 6:1 to 9:1, the yield was reduced. A higher methanol to oil ratio means that there is more methanol available to react with the oil, which leads to a higher yield of biodiesel [26]. However, if the methanol to oil ratio is too high, then the excess methanol will not react and will be wasted. The optimum methanol to oil ratio is typically between 6:1 and 9:1. At a constant stirring speed of 400 RPM and a constant 3 min reaction duration, it demonstrates the relationship between catalyst concentration, methanol to oil ratio, and percentage yield of biodiesel. At a 6:1 ratio, the highest yield of 95 % was attained. Fig. 5b depicts the connection between catalyst concentration, stirring rate, and yield. A higher catalyst concentration means that there are more catalyst molecules available to speed up the reaction, which leads to a higher yield of biodiesel [27]. However, if the catalyst concentration is too high, then the catalyst can become deactivated, and the reaction will slow down. The optimum catalyst concentration is typically between 1 % and 2 %.

**Fig. 6:**
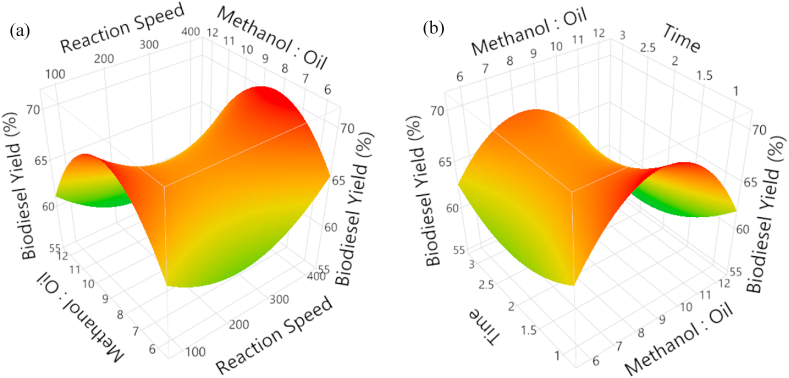
Experimentally obtained RSM plot to investigate the effect of (a) stirring speed, (b) reaction time, at a constant range of methanol to oil ratio.

The biodiesel yield peaked at stirring speeds between 180 RPM and 250 RPM and decreased at higher stirring speeds. Biodiesel production, the forward reaction is the transesterification reaction, where the oil molecules react with alcohol to form esters [28]. When the reaction speed is increased, the forward reaction is speed up, and the equilibrium point is shifted to the right. This means that more esters are produced, and the biodiesel yield increases. However, if the reaction speed is increased too much, the backward reaction can also be sped up. This can cause the equilibrium point to shift back to the left, and the biodiesel yield can decrease. Fig. 5c depicts the behavior of % yield with respect to catalyst concentration and reaction time with a constant methanol to oil ratio of 6:1 and a constant stirring speed of 400 RPM. A little increase in yield was observed when the reaction time was increased from 1 min to 3 min. The biodiesel yield was enhanced by increasing the catalyst concentration to 0.85 w/w. The trends are consistent with research results that have already been documented in the literature. Fig. 6a displays response surface plots as a function of methanol to oil ratio, stirring speed, and percentage yield of biodiesel at a fixed catalyst concentration and reaction duration of 1.5 w/w and 3 min, respectively.

The percentage yield increased from 70 % to 90 % by swiftly increasing the stirring speed from 250 RPM to 400 RPM with a methanol to oil ratio of 6:1. The maximum yield of biodiesel was created with a methanol to oil ratio of 9:1 to 10:1, as shown in Fig. 6b. Reaction time has an impact on yield as well.

**Fig. 7:**
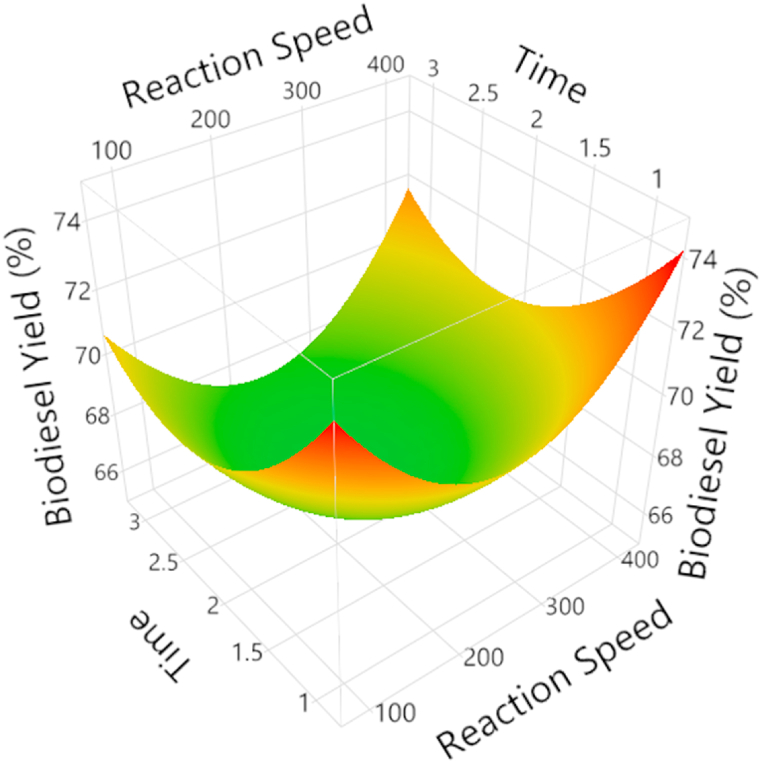
Experimentally obtained RSM plot to investigate the effect of reaction time at a constant range of stirring speed.

Fig. 7 shows surface response function of stirring speed, reaction time, % yield of biodiesel, and methanol to oil ratios of 1.5 w/w and 6:1 for a constant catalyst concentration. The graph demonstrates a consistent rise in yield as rotating speed and reaction time are increased as this has been discussed earlier. The backward reaction is the hydrolysis reaction, where the esters react with water to form the original oil molecules. When the reaction time and reaction speed increase, the concentration of water molecules increases, which speeds up the backward reaction [29].

The equilibrium point is the point at which the forward and backward reactions occur at the same rate. When the reaction time and reaction speed increase, the equilibrium point is shifted to the left, meaning that more of the original oil molecules are produced and less biodiesel is produced. When the reaction time and reaction speed increases, the esters can be converted to soap. Soap is a byproduct of the transesterification reaction, and it can reduce the yield of biodiesel [30].

### 3.5 Artificial neural network (ANN)

**Fig. 8:**
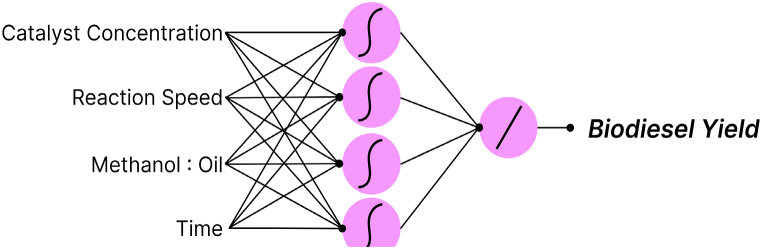
ANN model for Biodiesel Yield prediction.

Artificial Neural Network (ANN) is a function modeling technique. With enough data and right parameter selection any function may be modeled with this technique [31]. In ANN, artificial neurons are used to form a layer based on the number of variables [32]. The important parameter of a neuron is its activation function, there are a number of activation functions available [33]. The list includes linear activation functions, gaussian function and tangent hyperbolic function. There are more functions but only three were analyzed for ANN modeling of the yield. Several variations were tested. The dataset was split into a training set and validation set. Seventeen data points were used in training and six for validation. The transform covariate function was used to create higher dimensional data. The best results were obtained with a two-layer feedforward network with the first layer consisting of tangent hyperbolic activation function and the second layer with simple linear function. The use of the tangent hyperbolic function confirms the quadratic nature of the parameters predicted by the RSM. Fig. 8 illustrates the ANN model used for the prediction of the biodiesel yield.

Table 6 shows the R^2^ and standard deviation values for training and test set. The R^2^ value is close to one for the training and is 0.944 for validation. Thus, it confirms a well-defined model based on the training and validation dataset.

**Table 6:** Statistical analysis.

Measures	Training	Validation
R^2^	0.9999914	0.9440547
RASE	0.0274905	1.7084576
MAD	0.0214392	1.1267278
Loglikelihood	−36.9746	17.590766
SSE	0.0128474	26.269447
Sum Freq	17	9

**Fig. 9:**
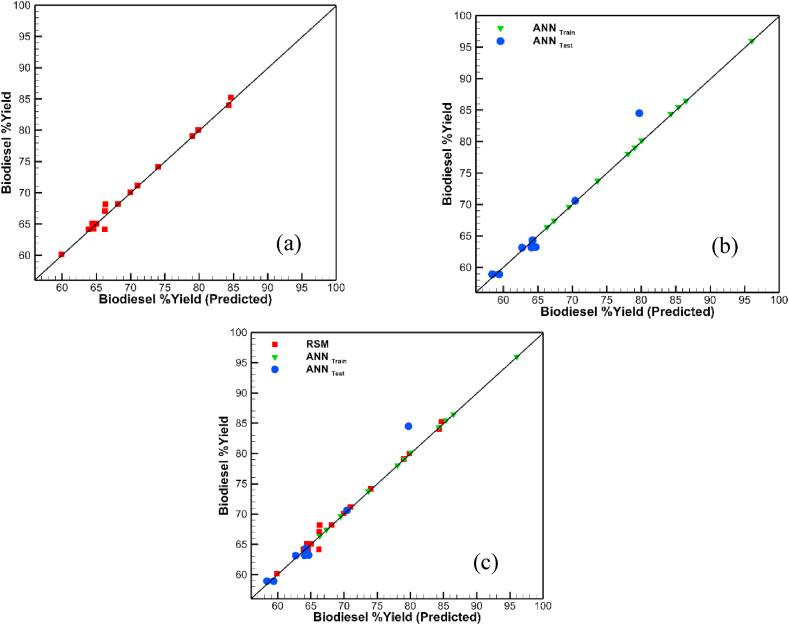
Biodiesel yield predicted validation models (a) RSM model (b) ANN training and test (c) Combined RSM and ANN models.

Fig. 9a, b, c revealed the comparison between the actual yield and predicted yield for the training and validation stages of the RSM and ANN models. All the points on the training section are on the direct relation line of slope 1. Thus, making a good training prediction. The optimization was applied on the ANN, to predict the maximum yield value of 95 % with parameter values of 1.5w/w, 400 RPM, 6v/v and 3 min for catalyst concentration, reaction speed, methanol: oil and time respectively. Moreover, this is the actual value from the experiment as compared to that of 88 % prediction by RSM.

### 3.6 Comparison of RSM and ANN models

The RSM and ANN techniques are applied for the biodiesel yield estimation. It is found that RSM is better at describing the interactions of different parameters with yield. RSM is also able to predict the yield as well. The interaction relations by RSM are like that of original behavior found in the experiments. However, upon comparison with RSM and ANN, the prediction of ANN is close to actual results. ANN is very sensitive to data as a supervised learning method of training and validation has been used. The presence of outliers in the data has to be removed in order for ANN to work properly. Secondly, if the model is over trained, it may predict the value of yield greater than 100 %, that too is a problem of overfitting. Therefore, for the application of ANN, a subject matter expert is required. With a well-trained model, the yield can be predicted with higher accuracy as compared to RSM. Therefore, both are used as their merit superimpose, RSM better describes interactions of individual parameters and ANN works better for overall yield prediction.

## 4 Conclusion

At the best operating circumstances, the yield of biodiesel was optimized. CBO FFAs were decreased by acid treatment. H_2_SO_4_ was shown to be the most efficient mineral acid. The FFA's value decreased by 90.4 %. It was discovered that using methanol to *trans*-esterify CBO was particularly successful. With a catalyst concentration of 1.5 %, a methanol to oil ratio of 6:1, a stirring speed of 400 RPM, and a reaction period of 3-min, 95 % biodiesel yield was achieved. The comparison of two statistical methods is done. The RSM predicts the interactions whereas ANN predicts the yield in the vicinity of the experimental results. Thus, the merits of RSM and ANN for biodiesel production is highlighted. Therefore, the production parameters may be optimized using these techniques.

## Data availability statement

The data is included in the article.

## CRediT authorship contribution statement

**Rehman Akhtar:** Writing – original draft, Data curation. **Ameer Hamza:** Data curation. **Luqman Razzaq:** Writing – review & editing, Writing – original draft, Supervision, Conceptualization. **Fayaz Hussain:** Conceptualization. **Saad Nawaz:** Software. **Umer Nawaz:** Software. **Zara Mukaddas:** Visualization, Data curation. **Tahir Abbas Jauhar:** Writing – review & editing, Writing – original draft, Visualization, Validation. **A.S. Silitonga:** Software, Resources. **C Ahamed Saleel:** Software, Resources, Funding acquisition.

## Declaration of competing interest

The authors declare that they have no known competing financial interests or personal relationships that could have appeared to influence the work reported in this paper.
